# Resuming hip and knee arthroplasty after COVID-19: ethical implications for wellbeing, safety and the economy

**DOI:** 10.1177/1120700020941232

**Published:** 2020-07-07

**Authors:** Nanne P Kort, Luigi Zagra, Enrique Gomez Barrena, Reha N Tandogan, Martin Thaler, James R Berstock, Theofilos Karachalios

**Affiliations:** 1 CortoClinics, Schijndel, The Netherlands; 2 IRCCS Istituto Ortopedico Galeazzi, Hip Department, Milan, Italy; 3 Department of Orthopaedic Surgery and Traumatology, Hospital La Paz, Autonomous University of Madrid, Madrid, Spain; 4 Ortoklinik and Cankaya Orthopaedics, Ankara, Turkey; 5 Department of Orthopaedic Surgery, Medical University of Innsbruck, Innsbruck, Austria; 6 Department of Orthopaedics, Royal United Hospital Bath, Bath, UK; 7 Orthopaedic Department, University General Hospital of Larissa, School of Health Sciences, Faculty of Medicine, University of Thessalia, Thessalia, Greece

**Keywords:** Coronavirus disease 2019, COVID-19, coronavirus 2, economic, ethical challenge, healthcare, orthopaedic, SARS-CoV-2, severe acute respiratory syndrome, total hip arthroplasty, total knee arthroplasty, revision arthroplasty

## Abstract

Reinstating elective hip and knee arthroplasty services presents significant challenges. We need to be honest about the scale of the obstacles ahead and realise that the health challenges and economic consequences of the COVID-19 pandemic are potentially devastating.

We must also prepare to make difficult ethical decisions about restarting elective hip and knee arthroplasty. These decisions should be based on the existing evidence-base, reliable data, the recommendations of experts, and regional circumstances.

## Introduction

A survey on behalf of the European Hip Society and the European Knee Associates has shown a massive reduction in primary and revision hip and knee arthroplasty surgery across Europe in response to the pandemic.^1^ Of the participating surgeons, more than 90% stated that their institutions no longer provided primary total joint arthroplasty. This reduction of arthroplasty services in Europe will have a detrimental impact on our patients’ pain, mobility, social life, and general health including cardiovascular wellbeing.^2–6^ Delaying the reintroduction of arthroplasty surgery will result in less favourable outcomes following surgery. Due to the reallocation of resources for COVID-19 patients, the ethical issue becomes: How long non-COVID-19 patients scheduled for elective orthopaedic surgery should be excluded from medical care? Elderly patients with multiple comorbidities scheduled for total hip arthroplasty (THA) or total knee arthroplasty (TKA) are at a higher risk of succumbing if infected with COVID-19 perioperatively, and may also require inpatient recovery in rehabilitation units or nursing homes further increasing the risk of transmission.

Total joint arthroplasty generates significant revenue for medical care centres, implant companies, and makes up a substantial portion of the daily income for arthroplasty surgeons. The projected value of the global overall joint market is $20.2 billion by 2025.^7,8^ These economic factors will influence the decision to re-start elective total joint arthroplasty during the COVID-19 pandemic. Thus, because our judgement will be affected by complex medical and economic factors, this article explores the five crucial ethical challenges to the resumption of THA and TKA after the COVID-19 pandemic in Europe.

## Patient safety

There are a few different categories of postponed patients waiting for total joint replacement during the pandemic.^9^ The first group, whose hip/knee disease severely affects their independence and wellbeing, are too concerned about disease transmission to seek medical attention. The second group of patients with joint conditions and risk factors for complications or death from COVID-19 are eager to undergo surgery, possibly not realising the potential risks and possible adverse outcomes which we cannot fully evaluate due to a lack of evidence. A third group which possibly includes half of our patients have joint conditions that would benefit from surgery but are unsure about proceeding in current circumstances and request guidance from the surgeon.

Patient safety is of utmost importance in guiding an ethical re-opening of our total joint arthroplasty services. Complications related to total hip and knee arthroplasties are well known to every surgeon, and adequately discussed at any informed consent procedure. However, the consent to elective surgery at the point when the pandemic is decreasing but cases are still being diagnosed requires a different kind of discussion. Most hospitals have incorporated specifically informed consents to add to those required for surgery. This specific, informed consent (IC) for elective surgery in times of COVID-19 requires further discussion with the patient about higher risk of virus transmission including from healthcare workers, the long incubation period (up to 14 days), the variable nature of the disease from mild to fatal, and discussions regarding ceilings of care and the potential need for ventilation. IC needs to clarify the patient’s understanding of these factors, and the specific measures taken by hospitals and staff to mitigate against each. Usually, those measures include prior testing of surgeon and staff, patient epidemiological interrogation and SARS-CoV2 testing (serology and/or PCR) before surgery (preferably 48–72 hours and no more than 7 days before surgery), and specific hospital pathways for non-COVID patients where patients may be protected to some extent.^10,11^ Of note, guidelines should be carefully tracked as consensus evolves along with the pandemic. Of course, a PCR positive patient on an elective pathway should be postponed. A quarantine of 14 days is recommended until the PCR becomes negative.^10,11^ Specific patient comorbidities are associated with a poor outcome following COVID-19.^10,11^ Among those, careful attention should be paid to severe cardiac conditions, diabetes, chronic lung disease, chronic kidney disease, immunocompromise, liver disease, severe obesity and age >65 years. Also, the emerging risk of thromboembolism related to COVID-19 means that consideration should be given to thromboprophylaxis regimes.^12^ Physical distancing, hand washing and use of masks must also be required at the hospital, and limiting the visits of relatives (1 single relative if required) is also part of this safety awareness.

## Patient prioritisation

The demand for arthroplasty is likely to exceed available resources after the resumption of elective surgical procedures. This demand may be exacerbated by reduced theatre productivity because of precautions used for the safety and protection of the patient and surgical team, limited availability of beds in intensive care units and hospital wards and limitations set by hospital administrators or health authorities. Unlike trauma cases, most patients needing arthroplasty are older and have associated comorbidities and therefore a higher risk of morbidity & mortality following COVID-19 transmission.^13^ This presents a dilemma for arthroplasty surgeons prioretising patients for arthroplasty surgery. Most guidelines at the peak of the coronavirus pandemic focused on emergency procedures such as periprosthetic fractures and acute infection or reconstructive arthroplasty after sarcoma resection as priority surgery and advised the postponement of other non-urgent joint reconstruction. With the resumption of elective surgery, several other guidelines have been published. The American College of Surgeons describe hip dislocation, knee dislocation, periprosthetic fracture, acute pain exacerbation in prior joint arthroplasty, inability to bear weight on the extremity, wound drainage, fever and concern about periprosthetic infection as priority indications for hip and knee arthroplasty surgery.^14^ The International Consensus Group and the AAHKS Research Committee recommend priority surgery for impending fracture and exposed implants, in addition to the conditions outlined above.^15^ The MeNTS Score (Medically Needed Time-Sensitive Procedures Score) takes procedural factors (overall procedure time, blood loss, need for intensive care unit, intubation probability); disease factors (viability of non-operative treatment, increased surgical difficulty and risk due to delaying the procedure) and patient factors (age, cardiopulmonary disease, diabetes, influenza-like symptoms and recent exposure to a known COVID-19-positive person) into account.^16^ This score can range from 21 to 105, with higher scores being associated with poorer perioperative patient outcomes, increased risk of SARS-CoV-2 transmission to the health care team and/or increased use of hospital resources. However, there is no threshold for safe elective surgery, and hospitals can adjust their thresholds depending on COVID-19 prevalence in their region and available resources. The International Consensus Group (ICM) and the AAHKS Research Committee recommend delaying elective surgery for patients over 75 years old with cardiopulmonary comorbidities, patients with morbid obesity, transplant patients undergoing immunosuppression and patients with active cancer.^8^ ESSKA guidelines advise giving priority treatment to younger patients (<60), requiring fewer than 3 days of hospitalisation and delaying elective surgery for patients with comorbidities.^17^ The European Hip Society and ESSKA-European Knee Associates are also working on joint recommendations on resuming elective hip and knee arthroplasty.

Although a variety of recommendations are available, the decision to select patients for arthroplasty ultimately rests on the shoulders of the surgeon. Factors not mentioned in the guidelines are; severe deterioration in quality of life, inability to weight-bear, sustained absence from work, dependence on assistance with activities of daily living, severe disease and deformity. The surgeon should weigh the relative benefits and risks of surgery, taking into account patient and disease factors, availability of resources and public health concerns, before they decide to offer surgery. This selection process should be fair, compassionate and free from financial concerns.

## Patient perspective

As orthopaedic surgeons, we should continue to treat our patients with honesty, compassion, skill and care. Our aims should always be to ‘cure and to care’.^18^ If we rely solely on technique and neglect our ethics of service, we become a trade and not a profession.^19^ The therapeutic alliance between doctor and patient should be based on understanding, confidence and cooperation and form the platform for a successful treatment.^20^ This quotation from the Ethical Orthopaedics for EFORT (European Federation of National Associations of Orthopaedics and Traumatology) has even greater value in this particular time of COVID-19.^21^ Postponing hip and knee arthroplasty may increase functional limitations and eventually result in loss of independence for many patients. This may also have an impact on a patient’s ability to survive in isolation or in difficult social circumstances. On the other hand, we have to accept that hospitalisation for hip and knee arthroplasty represents greater risk than previously, particularly in older patients with comorbidities. At the time this paper was written, most European health systems were beginning to recover from the pandemic.

If surgeries have been cancelled or postponed, waiting lists will grow, and there may also be limited availability for consultation services and face to face meetings with healthcare professionals. From a patient’s perspective, communication plays a significant role. The individual patient’s needs should be the focus of the doctor.^22^ Information is needed about treatment options while waiting for surgery, the risks of medication misuse, types of physical activity which could be beneficial for the individual patient, the evolving situation in the hospitals and the estimated time before intervention. This type of communication cannot be delegated to administrative staff at present.^23^ We have all recognised the potential of telemedicine as a tool for remote communication and patient evaluation. The challenge is to align with our patients’ expectations, and enable them to work with their surgeon.^24^ Shared decision making with full informed consent oriented explicitly to specific COVID-risks and issues must be considered. Some patients who are afraid spontaneously postpone surgery; they must be adequately informed about the risks and benefits of such a decision related to the specific COVID-19 situation at the time, and of preventive measures, including the need for preoperative screening. A case-by-case evaluation is necessary, but this can be time-consuming for the surgeon. A similar situation is the interaction with relatives as they are not permitted in the hospital. Ward rounds should incorporate remote communication, including daily phone calls with relatives of the hospitalised patients.^23,25^ Rehabilitation time is also problematic due to the lack of facilities including at-home services while admissions for rehabilitation are restricted to the minimum even for the older population, if not suspended. Therefore, careful ethical evaluation is required at an individual centre and for a specific patient, keeping in mind and discussing the pros and cons of early discharge.

In this challenging time, when reinstating elective surgery in a risky scenario with limited resources, surgeons have the responsibility to follow a shared decision-making process with the patient that includes an understanding of the legal aspects of complications, and COVID-19 specific, informed consent. At the same time, surgeons cannot ignore the most difficult cases in order to avoid any professional risk: this is probably the main ethical challenge in phase 2.

## Economical challenge

*‘Money is like blood – it needs to circulate for local economies to survive.’*

The COVID-19 pandemic constitutes an unprecedented challenge with very severe socio-economic consequences.^26^ The proposal for a Coronavirus Response Investment Initiative was approved by the European Parliament and the Council and is in force as of 1 April. This approval will allow the use of EUR 37 billion under the cohesion policy to address the consequences of the COVID-19 crisis. Also, the scope of the Solidarity Fund was broadened to include major public health crises. Starting from 1 April, this allows the hardest hit Member States to get access to the financial support of up to EUR 800 million that has been made available in 2020.

The global joint arthroplasty devices market is projected to exceed $20.2 billion by 2025, growing at a CAGR of 4.6% over the forecast period, driven by technological advancement and higher preference for and adoption of minimally invasive surgeries worldwide. Indeed, the demand for joint arthroplasty devices is expected to double within ten years, driven by robotically assisted operations, ageing populations, improvements in surgical and pain management techniques and moderate incremental innovations.^27^ Since 2000, the number of hip and knee arthroplasties has increased rapidly in most OECD countries. On average, hip arthroplasty rates increased by 30% between 2007 and 2017 and knee arthroplasty rates by 40%. This increase aligns with the rising incidence and prevalence of osteoarthritis caused by ageing populations and growing obesity rates in OECD countries.^28^ Without elective hip and knee arthroplasty procedures, our patients are at risk of increased pain and less mobility, and our health care institutions are at risk of insolvency. Patient risks derived from the lack of elective hip and knee arthroplasties include less independence due to joint pain or even joint destruction, which may also impact the ability to survive in isolation or under difficult social circumstances. Moreover, there is an increased risk of medication abuse by suffering patients.

Unfortunately, COVID-19 has had a tremendously negative impact on economic growth in 2020.^29^ Hospitals are on the front line and vulnerable to this economic disruption as they face challenges and hits to their revenue from the cancellation of elective surgeries. Most non-COVID-related activity has been halted due to the urgent demands of infected patients. As a result, health care providers are experiencing a significant reduction in revenue, while at the same time seeing increased staff and supply costs. Moreover, hospitals are unlikely to see ongoing contributions from non-operating income because their investment portfolios have been hurt, as well. Even before the coronavirus outbreak, many health care providers were struggling financially. The orthopaedic industry has also been witnessing a loss of business with some companies facing financial problems before the pandemic. Many orthopaedic companies have pro-actively planned for a worst-case scenario and reset their budgets to protect employees, customers and investors. The overall effect of the pandemic is impacting the production process of life science industries. Hip and knee arthroplasty deferrals and late resumption of the procedures will lead to revenue declines. There is a boom expected in hip and knee arthroplasties in the second half of 2020 once these procedures can be restarted, and revenue will once again be generated from such surgeries.^30^

An ethical discussion awaits us: how far do we allow the safety of patients and staff to prevail, and at what stage do we allow the economic side of this discussion to prevail?^31^ Above all, which is the safest, most effective way to treat our patients suffering from a joint disease at this time? The circumstances are different in every country, with a disparate impact of COVID-19 on the population and on health care providers. We need to find the right balance between medical safety and economic security. In any case, the decision to treat must not be based on financial reasons. In both privately and publicly funded systems, the decisions about the form of treatment that is offered should be based on need and not on finance.^31^ One thing is sure, with the downward trend in COVID-19 cases and deaths, there has been more and more focus on its economic impact, with tremendous pressure to restart primary hip and knee arthroplasties across Europe. At the same time, pressure from patients to be operated on soon is growing as they begin to feel safer about the path of the pandemic. In times of financial restraint, we know the problems caused when cost savings are achieved at the expense of patient care.^23^ There must be a balance between the risks and safety for our patients/staff and the economic pressure to restart the arthroplasty business.

## Public Health Care providers versus Private Health Care providers

In some countries, most arthroplasty surgeries are performed in public hospitals, while in other countries high volume arthroplasty surgeons work in private settings. Sometimes a combination of both is the preferred choice for arthroplasty service in a distinct region. Therefore, a general statement on a COVID-19 pandemic related shift of arthroplasty patients from one institution to another to reduce waiting lists and to satisfy the overall demand for arthroplasty is difficult. There is also a high variation in costs and reimbursement for total joint arthroplasty between countries,^32^ and therefore cross-country comparisons are difficult. Total joint arthroplasty is a frequently performed elective surgery and part of social benefit policies in many European countries; there is thus a significant budget impact for hospitals or private doctors.^33^ In recent years, countries with tax-based universal healthcare systems have experienced increasing attention from private healthcare providers.^1^ However, the difference in the quality of care is reported to be equal between public, private non-profit hospitals and private for-profit hospitals.^34^

Following general social distancing principles, reallocation of treatment of elective patients into a private sector might reduce the risk of SARS-CoV-2 infection of elective patients, because public hospitals or academic centres are more often confronted with COVID-19 patients. As well as potential additional costs for these patients or their health care providers, a patient shift to the private sector also might impair the education of the next generation of orthopaedic surgeons and science in general. It has already been reported that the COVID-19 pandemic has had a significant impact on the education and training of young surgeons.^35^ Also, the pandemic is currently disrupting clinical trials all over the world.^36^ Before the pandemic, most clinical science was performed at public, academic centres. Hence, a shift from elective joint arthroplasty patients from public hospitals into private hospitals would further disrupt clinical science and researchers and research questions might not be able to have direct contact with patients.

From an ethical point of view, the overall goal in the COVID-19 era is to provide protocols to safely perform hip and knee arthroplasty, irrespective of the set-up and environment. Arthroplasty should be performed in an environment where resources, staff and supplies can guarantee the patient’s safety. This can either be done in the private sector or public hospitals. There might also be a shift to ambulatory surgery centres, a public or private speciality hip and knee hospital, or fast track total joint arthroplasty in future. However, a transition during the pandemic will be challenging because all stakeholders will have to be convinced, and it has to be affordable for the patient. Our primary duties as orthopaedic surgeons are to serve our patients and reduce the risk of a second peak of COVID-19 cases even in times of long waiting lists and increasingly expensive procedures.

## Experience from different European countries

### Netherlands

The Dutch Hospitals’ Association, draws attention to the financial consequences of the COVID-19 epidemic for hospitals. Hospitals are confronted with higher costs and lower healthcare turnover. The care of COVID-19 patients and the adjustments in the outpatient clinic at the start of regular care cost money. As a result of the downscaling of regular care, income fell by 47%. In March, April and May, this may amount to approximately 2.1 billion euros. In addition, hospitals face additional costs in providing care for COVID-19 patients. For example, hospitals had to expand the number of IC beds, train or retrain other healthcare professionals, and continue to invest in digital care and purchase equipment and protective equipment. It is estimated that the extra expenditure in recent months is approximately 0.5 to 2 million euros per month per hospital and may rise to nearly 3 billion euros in the coming years.

### Italy

Italy was the first country forced to face the COVID-19 emergency after China. The emergency has put a strain on the health system, both for the rapidly increasing need for intensive care unit beds and for the growing number of patients suffering from less severe disease that needed to be treated in the hospitals. All elective procedures have been stopped during the pandemic, only infections, oncology cases and acute trauma were treated in a network system at regional level where a few hubs were identified for orthopaedic and trauma urgent cases while general hospitals were taking care of COVID-19 patients. During the second half of April and May the situation was improving and elective surgery is slowly increasing. Nevertheless the demand from health authorities is to operate on patients younger than 70 with few comorbidities that are on a priority list, and not more than 60–70% of the volume done in the same period of 2019. Hospitals must remain ready for a rapid conversion to COVID-19 care in case of a second wave.

### Greece

The first COVID-19 patient was diagnosed in Greece on the 26th of February 2020. A complete lockdown of the country was implemented on March 11th. As a result, all elective Orthopaedic Surgery was halted (both state and private sectors) and only musculoskeletal trauma, infection, and Orthopaedic Oncology were dealt with.

On Monday, May 4th a restart of surgical procedures was enacted at a level of 50% (predominantly musculoskeletal trauma) of hospital capacity, while taking strict preventive measures. Despite satisfactory clinical and social management of the pandemic, there has been a serious impact on elective Orthopaedic services with broad ethical and social implications. In a country with a yearly average of 20,000 primary and revision implant surgeries, a very small number of cases are now performed. Waiting lists have increased and patients are now expected to endure symptomatic joint disease and resulting disability for an indeterminate length of time. Implant providers have also seen revenue reduced to 20%, and despite the fact that their employees have been included in a partial unemployment scheme supported by low income state benefits, it is expected that job losses will be recorded at the level of 50%.

### Austria

Comparing international data, Austria is ranked among the top countries with respect to its population-based implantation rate of 210 per 100,000 for total hip arthroplasty (THA), and 202 per 100.000 for total knee arthroplasty (TKA). Austria was considered 1 of the hotspots for the COVID-19 outbreak at the beginning of the pandemic In Europe. Hence, the countries lockdown was on March 14th, including some parts of the country being under quarantine and stopping all elective surgery on March 16th. However, after the COVID-19 curve flattened, approximately 50% of elective arthroplasty volume was started at April 12th, followed by full resumption at May 11th.

### Turkey

90,000 hip and knee arthroplasties are performed annually in Turkey, with a market of 55 million Euros in implant costs. This amounts to 1/6 of the entire orthopaedic implant/consumables market of the country. With the identification of first COVID-19 cases in 14 March 2020, select hospitals were designated to treat COVID-19 patients, however all elective orthopedic surgeries were halted in other hospitals to provide a back-up for overflowing cases from COVID-19 hospitals. This led to a 98% cessation of arthroplasty procedures until June 1st. With the down slope of the pandemic curve, elective surgery will start at 50% volume on June 1st, followed by full resumption on June 15th, 2020 if no surge in COVID-19 cases occurs.

### Spain

Currently (26 May 2020), Spanish elective total joint replacement surgery is timidly opening. In late May, joint reconstructive surgery has been restricted in most tertiary hospitals to infections, particularly 2-stage revision surgery. Hospitals have incorporated defined protocols to assess serology and SARS-CoV2 PCR in surgeons and staff, but also in every patient scheduled for any surgical intervention. Furthermore, ICU needs after surgery are planned, besides regular postoperative care, while spinal or epidural anaesthesia was already the standard for hip and knee procedures in many hospitals. Although the number of operating rooms available for scheduled orthopaedic surgery lies at 50%, the number of available hospitalisation beds and ICU beds is within the required limits, and low risk patients are already selected to start total knee and hip replacement this week. A careful monitoring of each institution is required, and the impact of the clockstop for elective joint replacement surgery will probably endure until the end of the summer. Meanwhile, elderly patients with comorbidities are refusing to visit clinics. Safety needs improving and patients need support to regain confidence in healthcare.

### United Kingdom

In the UK, the National Health Service advised hospitals to postpone elective surgery on 17th March 2020 for 12 weeks to free up capacity for the increasing numbers of COVID-19 patients being admitted to hospitals. It is estimated that there have been a total of 516,000 postponed surgeries, including 36,000 cancer procedures. Private sector hospitals have been repurposed to help deliver urgent services, but planned joint replacement surgery has ceased throughout this period. Measures such as social distancing and self-isolation have resulted in falling numbers of COVID-19 cases in most parts of the UK, buying time to increase ventilator numbers and free up surge capacity within our hospitals during the first phase of our response to the pandemic. We are now entering the second phase where we are beginning to reintroduce elective surgery, including joint replacement.

The reintroduction of elective joint replacement during the COVID-19 pandemic poses greater organisational and ethical challenges than its cessation 12 weeks ago. Frameworks for the safe reintroduction of Orthopaedic surgery have been drawn up by NHS England, and the British Orthopaedic Association in mid May 2020.

There is consensus that 2 very separate pathways are required; 1 for COVID-negative planned elective work, and the other pathway for urgent or emergent care. We require planned-surgery candidate patients to isolate for 14 days and test negative on home COVID swab kits within 72 hours of admission. Currently strict isolation of all members of the household is required, however this will be challenging for most patients, and it may be similarly effective for just the individual concerned to isolate from other household members for 14 days. Either way, there are concerns that not all patients will comply, risking an outbreak in within a COVID-negative pathway.

Rules for the staff treating patients are currently being determined locally. It makes sense for staff to work exclusively within COVID-free pathways for periods of time with an interval before alternating from urgent to elective (COVID-free) pathways. Teamworking will be required among arthroplasty surgeons to provide alternating periods of planned joint replacement and urgent revision surgery for periprosthetic fractures and prosthetic joint infection.

Provisions will need to be made for outbreaks within the COVID negative pathways, with extreme vigilance and plans set out for immediate isolation of patients and staff with symptoms.

## Conclusion

The ethical tenant of ‘achieving the most good’ with limited theatre resources makes the reintroduction of non-urgent joint replacement an important milestone in the societal recovery from the pandemic. Doing good must be balanced with doing the least harm. We thereby have a duty to mitigate risk for our patients, and so it may be prudent to establish our pathways and processes for low risk patients such as the young undergoing day case orthopaedic surgery, before the reintroduction of joint replacement for more frail patients.

**Figure 1.**
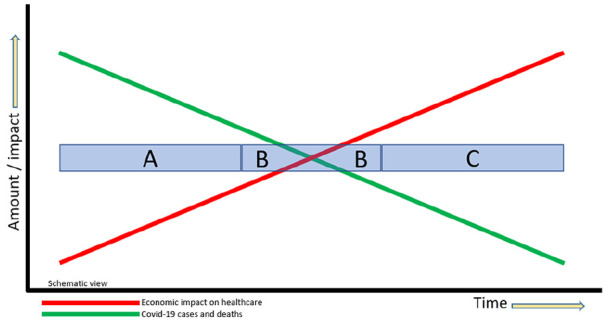
Trends in COVID-19 cases versus economic impact on health care. (A) Earlier start to hip and knee replacement may endanger patients and staff (B) Restarting hip and knee replacement at the right moment is the challenge (C) Later start to hip and knee replacement may jeopardise health care institutions.

We have seen a downward trend in COVID-19 cases and deaths yet the economic impact of COVID-19 on health care institutions, the orthopaedic industry and health care providers continues to rise. These factors will eventually intersect, depending on which country you live in (Figure 1). Restarting hip and knee replacement at this moment of intersection (area B) is the challenge. If we start up hip and knee replacements earlier (area A), we may endanger patients and staff. If we start later (area C), we may jeopardise health care institutions in an already fragile health economy.

The consent process must include making patients aware that despite efforts to minimise disease transmission, the risk of hospital-acquired COVID-19 cannot be eliminated. Patients will need to exercise their autonomy when deciding whether to come into hospital for planned surgery based on the most accurate advice we can give. Ultimately, many of our joint replacement patients are elderly and comorbid, living in pain. Some will even be enduring a quality of life ‘worse than death,’ and may wish to proceed despite the high risks of mortality from contracting COVID-19 in the perioperative period.

We will need a major catch up effort to avoid additional harm to our patients waiting in the backlog.
